# Supplementary material for: A Computational Model of Visual Recognition Memory via Grid Cells
Source: Curr Biol. 2019 Mar 18;29(6):979–990.e4. doi: 10.1016/j.cub.2019.01.077 (PMC6428694; doi:10.1016/j.cub.2019.01.077)
Supplement: Document S1. Figure S1 and Table S2 [file mmc1.pdf]

Current Biology, Volume *29*

Supplemental Information

A Computational Model of Visual Recognition Memory via Grid Cells

Andrej Bicanski and Neil Burgess

Supplementary Material

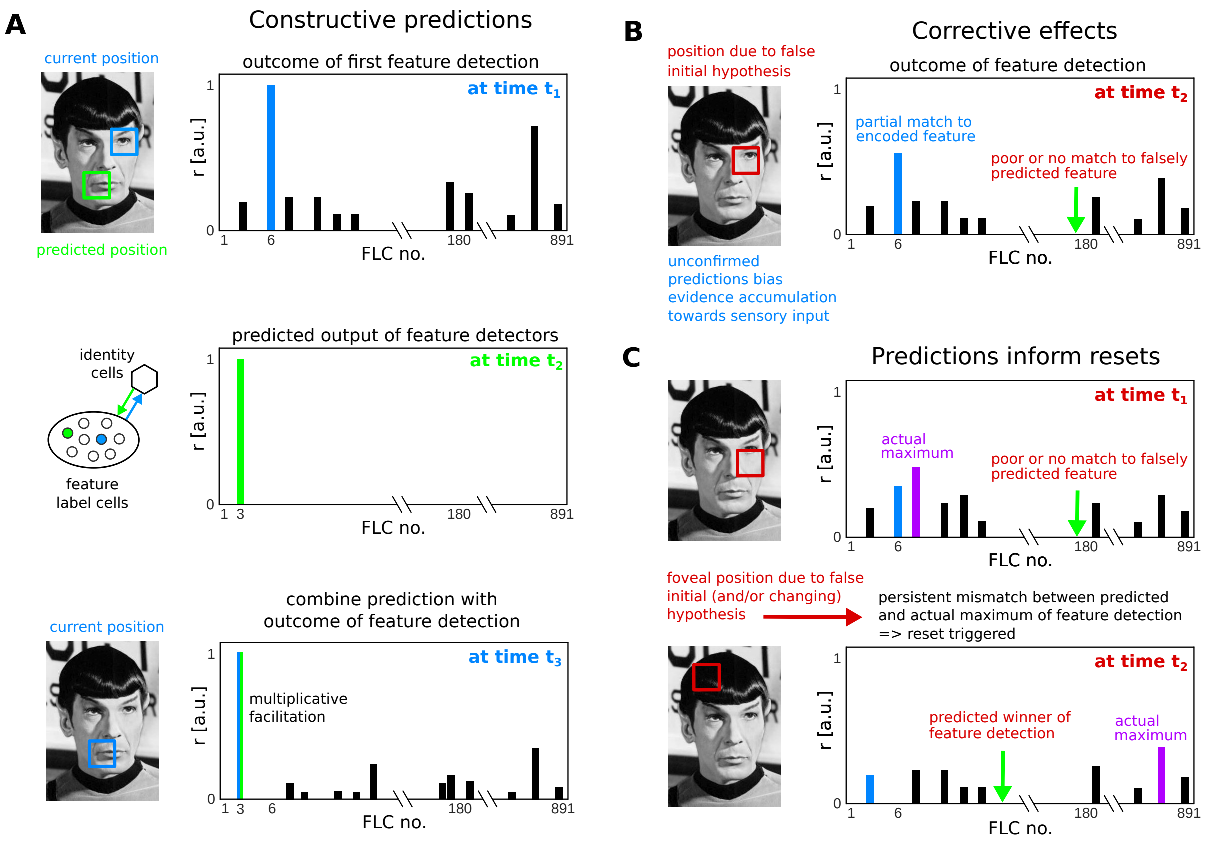

Figure S1: Detailed description of the effects of sensory predictions. (Related to Figure 1E)

A) The constructive nature of confirmed predictions. Top panel: The first foveated feature is evaluated by the feature detectors, which drive feature label cells (FLCs). The feature label cell representing the right eye of Mr. Spock is the strongest contender (blue bar in left sketch of FLC activity after thresholding), and it increments its associated stimulus identity cell (middle-right panel, blue arrow). The return projection (green arrow) selects the next saccade target randomly (green cell, target given by associated grid cell population vector, not shown). However, the firing of that FLC (green) also represents a prediction for the outcome of the next sensory discrimination once the fovea has located to the green square (top-left panel). Bottom panels: if there is any activity in the predicted FLC post saccade, its activity is boosted, allowing it to overcome interference from FLCs which received comparable or lower inputs. I.e. the leading hypothesis is prioritized if the competing inputs are of similar magnitude. **B)** Top panel: the lack of a confirmed prediction made due to an incorrect

initial hypothesis (green arrow in right panel) allows a partial feature match (blue bar) to accumulate evidence towards the correct hypothesis. **C)** If the predicted outcome of the feature discrimination (green arrow) and the maximally firing FLC (purple) do not match a mismatch event is registered. The red square in the left hand side panels indicates fixations to parts of the stimulus that will produce a poor sensory match due to being guided by an incorrect hypothesis. At the third registered mismatch event the model resets and starts a new recognition attempt, beginning with a different starting feature. Image credit; Mr. Spock: public domain image.

Table S1: All stimuli with picture credit (source URL) and license information. (Related to Results section and Figures 3,4,5)

In accordance with CellPress guidelines this table is supplied as a separate Excel file because it cannot fit onto three 8.5”x11” pages.

N_{std}	2.8
N_{Reset}	10
FLC2ID	0.4
Dec_{TH}	0.9
N_{fov}	61x61/31x31
N_{FPC}	9*99
N_{ID}	99
N_{SC}	61x61x256/31x31x256
N_{GC}	900 (100/module)
GC spatial frequencies	[0.0014 0.0211]
N_{DC}	440x4 (2 per axis)

Table S2: Main Parameters. (Related to detailed model description in STAR* Methods)

N_{std} sets the amount of initial pruning of feature label cell responses. It indicates the number of standard deviations the firing of a feature label cell has to reach above the mean of the entire population in order not to be silenced. N_{Reset} is the number of resets allowed. *FLC2ID* is the global connection strength of all feature label cells to their respective stimulus identity cells. The value of 0.4 signifies that if a single feature label cell is active (i.e. when the softmax across feature label cells has no effect), the firing of the associated stimulus identity cell is increased by 0.4 towards the decision threshold (Dec_{TH}). Cell numbers: Number of foveal cells (N_{fov}, second set of numbers indicates extent used for scaled-down stimuli), feature label cells (N_{FLC}), stimulus identity cells (N_{ID}), sensory cells (N_{SC}), grid cells (N_{GC}) across 9 modules across the interval (spatial frequencies geometrically spaced, multiplier $\sqrt{2}$), and distance cells (N_{DC}).
