# Supplementary material for: A Computational Model of Visual Recognition Memory via Grid Cells
Source: Curr Biol. 2019 Mar 18;29(6):979–990.e4. doi: 10.1016/j.cub.2019.01.077 (PMC6428694; doi:10.1016/j.cub.2019.01.077)
Supplement: Document S2. Article plus Supplemental Information [file mmc3.pdf]

Current Biology

Article

A Computational Model of Visual Recognition Memory via Grid Cells

Highlights

- Visual grid cells can encode saccade vectors between salient stimulus features
- A sequence of (memory-guided) saccades confirms stimulus identity
- Known properties of grid cells confer size and position invariance onto the model
- Grid cell lesions may impair relational memory and could contribute to prosopagnosia

Authors

Andrej Bicanski, Neil Burgess

Correspondence

a.bicanski@ucl.ac.uk (A.B.),
n.burgess@ucl.ac.uk (N.B.)

In Brief

Bicanski and Burgess propose that grid cells support visual recognition memory, by encoding translation vectors between salient stimulus features. They provide an explicit neural mechanism for the role of directed saccades in hypothesis-driven, constructive perception and recognition, and of the hippocampal formation in relational visual memory.

Bicanski & Burgess, 2019, Current Biology *29*, 979–990
March 18, 2019 © 2019 The Author(s). Published by Elsevier Ltd.
https://doi.org/10.1016/j.cub.2019.01.077

CellPress

Current Biology
Article

A Computational Model of Visual Recognition Memory via Grid Cells

Andrej Bicanski[1,2,*] and Neil Burgess[1,*]
[1]Institute of Cognitive Neuroscience, University College London, Alexandra House, 17 Queen Square, WC1N 3AZ London, UK
[2]Lead Contact
*Correspondence: a.bicanski@ucl.ac.uk (A.B.), n.burgess@ucl.ac.uk (N.B.)
https://doi.org/10.1016/j.cub.2019.01.077

SUMMARY

Models of face, object, and scene recognition traditionally focus on massively parallel processing of low-level features, with higher-order representations emerging at later processing stages. However, visual perception is tightly coupled to eye movements, which are necessarily sequential. Recently, neurons in entorhinal cortex have been reported with grid cell-like firing in response to eye movements, i.e., in visual space. Following the presumed role of grid cells in vector navigation, we propose a model of recognition memory for familiar faces, objects, and scenes, in which grid cells encode translation vectors between salient stimulus features. A sequence of saccadic eye-movement vectors, moving from one salient feature to the expected location of the next, potentially confirms an initial hypothesis (accumulating evidence toward a threshold) about stimulus identity, based on the relative feature layout (i.e., going beyond recognition of individual features). The model provides an explicit neural mechanism for the long-held view that directed saccades support hypothesis-driven, constructive perception and recognition; is compatible with holistic face processing; and constitutes the first quantitative proposal for a role of grid cells in visual recognition. The variance of grid cell activity along saccade trajectories exhibits 6-fold symmetry across 360 degrees akin to recently reported fMRI data. The model suggests that disconnecting grid cells from occipitotemporal inputs may yield prosopagnosia-like symptoms. The mechanism is robust with regard to partial visual occlusion, can accommodate size and position invariance, and suggests a functional explanation for medial temporal lobe involvement in visual memory for relational information and memory-guided attention.

INTRODUCTION

How the brain implements recognition of familiar faces, objects, and scenes at the neural level is a complex problem that has engendered a multitude of different approaches. Both unsupervised and supervised learning systems have been proposed for the classification of visual stimuli into categories, and for recognition of specific familiar stimuli within a category [1–3]. These approaches often focus on the parallel processing of low-level visual features, inspired by the landmark findings of Hubel and Wiesel [4], and on how higher-level representations emerge at later processing stages.

However, since the pioneering studies of eye movements by Yarbus [5], perception has been known to also depend on motor acts, which are necessarily sequential. The notion that sequences of saccades (rapid target-driven eye movements) might underlie complex pattern recognition and constructive, hypothesis-driven perception is an idea with a long history in neuroscience (e.g., focal attention and figural synthesis [6]; Scanpath theory [7, 8]), and has been taken up repeatedly since (e.g., [9]). It is also supported by rare pathologies in which patients incapable of performing eye movements emulate saccades with head movements [10]. According to this account, the currently attended part of a stimulus is foveated and used to calculate a saccade to where the next feature of the stimulus should lie according to the hypothesized stimulus identity. For example, on foveating the nose of a familiar face, the facial identity is confirmed by generating a saccade to where that person's left eye should be and, if detected, thence on to where their mouth should be, and so on. This implies consistency of saccade targets (though not necessarily order) between encoding and retrieval conditions, which is supported by behavioral data [11, 12], in particular, by memory-guided saccades, which depend on the return of gaze to previously encoded (i.e., fixated) locations [13, 14].

Decoupling depth perception (i.e., ocular focus) from yaw and pitch movements of the eye, a sequence of saccades can be viewed as a complex trajectory on a 2-dimensional plane (the field of view). This allows us to exploit analogies to studies of spatial navigation in which experimental animals move freely on a 2D plane while neuronal activity is recorded. These paradigms have revealed neuronal responses in and near the hippocampus that are well suited to represent locations and trajectories in two dimensions. In fact, so-called place cells, which exhibit firing specific to a single location in an environment [15], and grid cells, which exhibit multiple regularly arranged firing fields [16], have become cornerstones of our growing understanding of spatial cognition.

Several recent studies have suggested that entorhinal cortex cells can exhibit grid cell-like responses in visual space [17–20]. Here, we propose that these visually driven grid cells

This is an open access article under the CC BY license (http://creativecommons.org/licenses/by/4.0/).

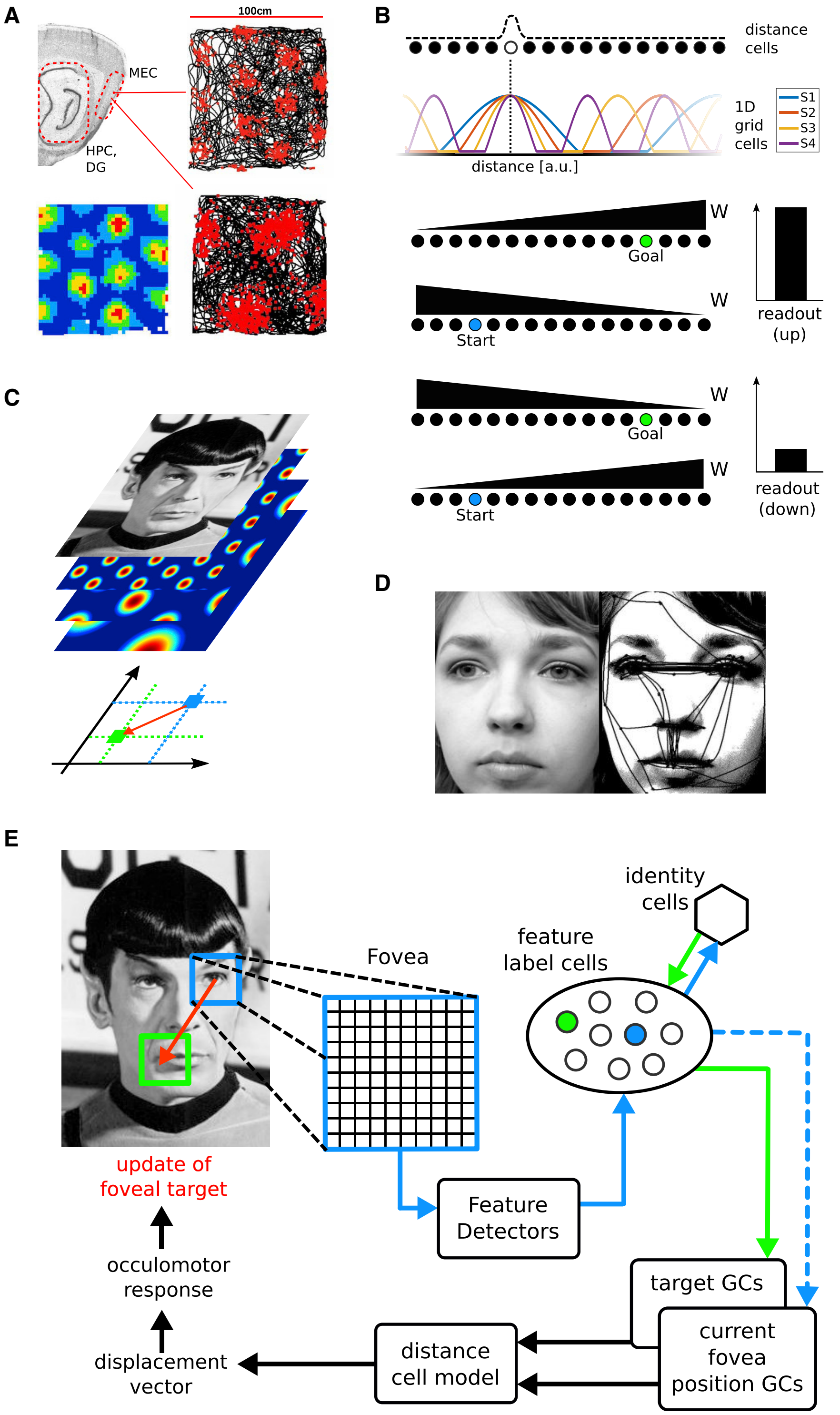

Figure 1. Grid Cell-Based Vector Computations and Visual Recognition Memory

(A) Grid cells in medial entorhinal cortex (MEC) exhibit periodic, hexagonally arranged firing fields, originally characterized as spatially selective cells in rodent experiments. Right: Spiking locations (red dots) superimposed on a rodent's trajectory during foraging; Bottom left: A stereotypical, smoothed firing rate map.

(B) Vectorial coding in one dimension. 1D grid cells with appropriate phases across modules (top: 4 cells with different scales S1–4) project to distance cells. Distance cell arrays (2 for each direction along the 1D axis) project with monotonically increasing weights (*W*) to two readout cells (for up and down, respectively). The difference in output between readout cells is a measure of the distance and direction between start (blue) and goal (green) locations.

(C) Replicating distance cells and readout cells for a second, non-co-linear axis allows computation of 2D vectors in the stimulus (e.g., between facial features, to guide eye movements, e.g., in D).

(D) A face with superimposed saccade trajectories.

(E) Model schematic: Grayscale images are sampled by a square fovea (blue square). Feature detectors drive feature label cells, each coding for a particular salient feature. During training each feature label cell has been associated with a grid cell population vector (current position grid cells, blue cell and dashed arrows). All feature label cells of a given stimulus are bi-directionally connected to a single cell coding for the identity of the attended stimulus. Upon firing of an identity cell the currently active feature label cell is inhibited and identity cells select the next feature label cell to be active (short green arrow and cell), which is associated with its own grid cell population vector (target grid cells). Current and target position grid cell representations yield the next saccade vector (red arrow on image). Note, the selection of the feature label cell corresponds to a prediction of the next sensory discrimination (see also Figure S1). Image credit: Mr. Spock: public domain image; Grid cell rate map and rodent trajectory adapted from Barry and Bush Neural Systems & Circuits 2012 2:6, Attribution 2.0 Generic Creative Commons CC-BY 3.0; Saccade trajectories reproduces from Wikimedia Commons; Attribution 2.0 Generic Creative Commons CC-BY 3.0).

support recognition memory by encoding inter-feature movement vectors, capturing the layout of compound stimuli in a stimulus-specific coordinate system. This model might also account for medial temporal lobe interactions with the visual system during visual recognition of relational information or object-location binding [21–24] and memory-guided attention [14, 15], and encoding of saccade direction within entorhinal cortex [25].

Grid cells have been suggested to provide a spatial metric that supports path integration (by integrating self-motion inputs [26]) and vector navigation [27–29]. Located in medial entorhinal cortex, grid cells form modules of different spatial scales (Figure 1A). Within a module, different offsets (or phases) are present and the firing fields of relatively few grid cells evenly cover an environment. The spatial periodicity of grid cells at different scales suggests that they provide a compact code for location. A set of phases of grid cells across multiple spatial scales can uniquely encode locations within a space much larger than the largest grid scale

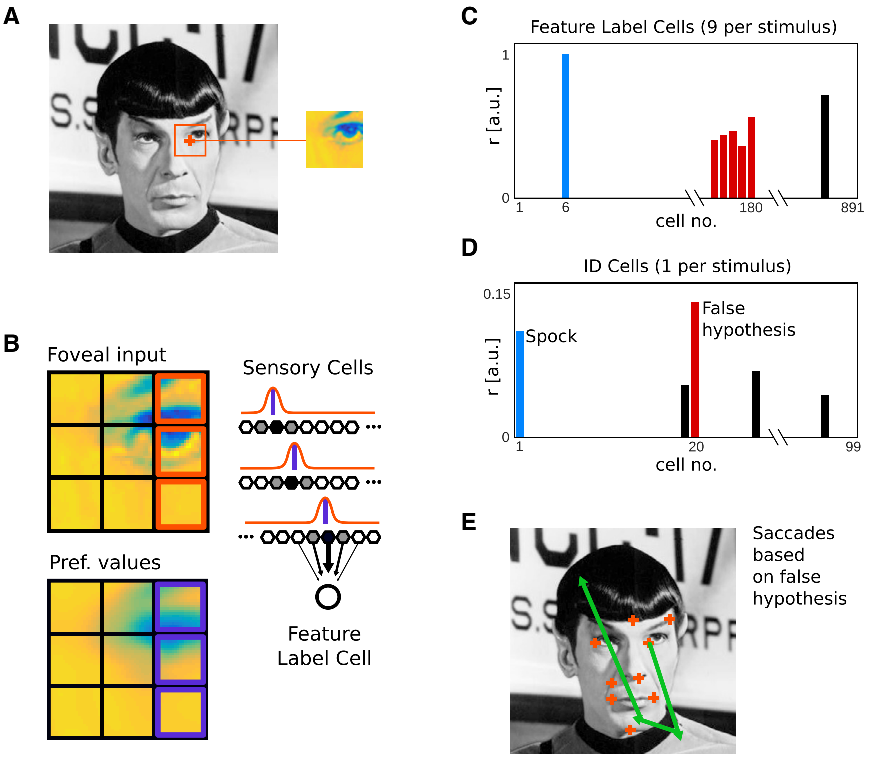

Figure 2. Feature Detection and Ambiguity

(A) The first (randomly chosen) stimulus feature is assumed to attract attention in a bottom-up manner.

(B) The foveated feature is compared to imprecise reference values by banks of sensory cells (hexagons). Each pixel drives a given set of sensory cells maximally (filled hexagons). Here, the responses for 3 pixels are illustrated (red Gaussians and pixels). During training, a blurred version of the stimulus is presented (purple pixels and bars [preferred values] under Gaussians), resulting in feature ambiguity. Connections between sensory cells and feature label cells are learned (black arrows, only one set of connections shown for clarity).

(C) Feature ambiguity could lead to a feature label cell from an incorrect stimulus identity being the most active, or multiple feature label cells from an incorrect stimulus being partially active (red).

(D) In either case, the corresponding (incorrect, red) stimulus identity cell will receive more input than the correct one (Spock, blue), and the system starts with an incorrect hypothesis.

(E) Thus, the incorrect stimulus identity cell (red) determines the next saccade(s), which cannot bring the memorized features of the stimulus into foveal focus. Image credit: Mr. Spock: public domain image.

[29] (see also STAR Methods). Several potential neural network architectures can be built to compute the direct vector between any two locations in 2D space. Here, we suggest that visually driven grid cells encode vectors between salient stimulus features in visual space to drive saccades in the service of visual recognition. Intriguingly, the known properties of grid cells can confer size and position invariance onto the model and help it deal with occlusions. The model suggests clear predictions, and the proposed recognition mechanism conforms to a large body of literature on visual recognition (in particular holistic face processing).

For a detailed description of the model, see the STAR Methods. Briefly, a population vector across the grid cell ensemble uniquely defines a position in the visual field (Figures 1A and 1B). Any two such positions can be used as inputs to a distance cell model [29], which yields the displacement vector between those two locations (Figures 1B and 1C). This vector calculation is integrated with visual processing in the following manner. Grayscale images (440 × 440 pixels) represent pre-processed visual input to the model. A small square array of cells represents output from a simplified fovea (61 × 61 cells) with associated sensory cells (feature detectors). In a training phase, the model learns Hebbian associations between the following cell types. First, as an individual component feature (e.g., the nose, eyes, lips, etc. of a given face) of a stimulus is foveated, it generates a characteristic response among the feature detectors (i.e., individual features are evaluated; Figure 2). A Hebbian association between the array of feature detectors and an individual (newly recruited) feature label cell (representing the foveated component feature of a stimulus) is then learned. Second, a connection between feature label cells and the locations of those features in the visual field (represented by grid cell population vectors) is learned. Finally, all feature label cells belonging to a stimulus are associated—bi-directionally—to a stimulus identity cell, representing the abstract identity of a stimulus. That is, each stimulus identity cell receives connections from a small number of feature label cells representing the component features of that stimulus and has a return projection to the same feature label cells (Figure 1E).

Once the model has learned the necessary associations, its recognition memory is tested by presenting stimuli from the training set. An action-perception cycle then consists of the following steps: the foveal array is centered on a given feature (we assume that the first feature is selected by bottom-up attentional mechanisms, which are not modeled here). Feature detectors (perception) drive the feature label cells, which (partially) match the attended feature. Thresholding and a subsequent softmax operation ensure a sparse code among feature label cells. Active feature label cells drive their associated stimulus identity cells, generating competing hypotheses about the stimulus identity. The most active identity cell then determines the computation of the vector for the next saccade (action) in the following way. The current location of the fovea is represented by a population vector of grid cell activity, which is updated by eye movements analogously to how grid cell firing is updated by self-motion during navigation [26]. This yields the starting point of the saccade vector. Previously active feature label cells (including the one for the currently foveated feature) are reset to zero, and the most active stimulus identity cell (representing the leading hypothesis) randomly selects the next feature label cell via its return projection. Randomness is given by weak noise on the back projection, and winner-take-all dynamics select

the feature label cell, which is in turn associated with its own grid cell population vector (yielding the end point of the next saccade vector). Given the starting and end points of the next saccade, the distance cell system outputs the vector required to update foveal position (see Figure 1E for an overview; details in STAR Methods), allowing the system to sample another portion of the visual field. The cycle then repeats while stimulus identity cells accumulate firing across cycles until a firing rate "decision threshold" has been reached (at which point the leading hypothesis about the identity of the stimulus is accepted and identity cells reset to zero before the next stimulus is presented).

Importantly, in addition to specifying the endpoint of the next saccade via associated grid cells, the activated feature label cell that has been selected by the return projection of the leading stimulus identity neuron also represents a prediction. Once the fovea relocates, and the next sensory discrimination is carried out, the maximally active feature label cell should be the predicted one. This prediction is incorporated as a facilitatory effect, boosting the firing of the predicted feature label cell in the next cycle by a factor of two, prior to the application of the softmax operation across all feature label cells. If the predicted feature label cell is not the most active one after the next sensory discrimination, a mismatch is registered. At the third mismatch event, the system resets, beginning with different component feature. This procedure allows for a fast rejection of false hypotheses, which will otherwise produce saccades that do not take the fovea to expected features. Figure S1 details the effect of sensory predictions.

During learning, each feature of a given stimulus is anchored to the grid cell representation, and the relative locations of features encoded across all grids are mutually consistent. Paralleling experimental results [17, 20] we encode all stimuli in the same grid ensemble (as if anchored to a presentation screen). However, if connections from the most active feature label cell can re-align the grid cell ensemble (foveal array ⇒ feature label cell ⇒ grid cells) to the phases specific to a given stimulus, then recognition irrespective of the position of a stimulus in the field of view (position invariance) follows from the fact that the grid system encodes the relative (rather than absolute) locations of the features within a stimulus. This is consistent with grid cell firing patterns being stable across visits to the same environment but shifting coherently across different environments [30]. This predicts that grid cell phases would follow the position of the stimulus in the visual field, in the absence of environmental anchor points, e.g., during recognition of illuminated faces in darkness.

Regarding size invariance, pre-processing of visual inputs could generate a size invariant representation prior to input to the system described here. However, the present model could accommodate size invariance. All circuit level models of grid cells require velocity inputs to update their firing (locomotor velocity during spatial navigation or eye-velocity in visual paradigms). The coupling between "neural space" (i.e., distance on the grid pattern) and self-motion has been shown to be plastic in spatial navigation paradigms [31]. Hence, we assume a given saccade length is subject to gain-modulation to give an appropriately scaled distance on the grid cell ensemble, and, conversely, a given distance on the grid cell ensemble can be scaled to yield a saccade of appropriate length. This requires a change in gain with the estimated size (distance) of the stimulus. The estimation of stimulus size during recognition could reflect the size of the segmented retinal image or ocular focus (compared to a memorized baseline), which is related to the distance and hence size of the stimulus.

RESULTS

Following one-shot learning trials (where the stimulus identity, feature label, and grid cell associations have been encoded; see Figure 3A), the model is tested on the stimuli it has learned. Once a starting feature has been selected (at random, in the absence of a model for bottom-up attention), grid cell-driven recognition memory takes over, calculating saccade vectors to predicted locations of other stimulus features. The firing of identity cells in response to the first perceived feature signifies the generation of hypotheses about the stimulus being observed, and the leading hypothesis (i.e., the most active identity neuron) determines successive saccades to confirm that hypothesis (Figures 3B–3D). Each saccade represents an attempt to move a different part of the stimulus into foveal focus, based on the leading hypothesis (e.g., attending the eyebrow of Mr. Spock, moving the eyes in a certain direction and distance, should bring his nose into focus). Once a stimulus identity cell reaches the threshold for recognition, the next stimulus is presented. Figures 3B–3D show examples of successfully recognized stimuli. In Figure 3B, panel 3 (Earhart), the system started off with a wrong hypothesis (black arrows in line plot) but subsequently recovered. Because the relative arrangement of features can be similar between the competing stimuli, successive saccades (relative displacement vectors) eventually let the correct stimulus identity accumulate more evidence because of partial matches with encoded features. That is, the model does not rely exclusively on resets if the initial hypothesis is wrong. The correct hypothesis can overtake an initially leading, incorrect hypothesis. A reset on the other hand is illustrated in Figure 3C, panel 2 (Dido Building Carthage) where the initially incorrect hypothesis led to more than two mismatches between the expected and actual outcome of feature discrimination and hence triggered an early reset. On the second try, starting with a different feature the system recognizes the stimulus within 5 fixations. Figures 3B–3D show examples for face, scene, and object stimuli, respectively. A total of 99 stimuli were tested (33 faces, 33 scenes, 33 objects; see Table S1).

Most stimuli are recognized within 4–6 saccades (from last reset; Figure 3E), and 98 out of 99 stimuli were successfully recognized (see Figure 5G for summary statistics). These numbers reflect the amount of thresholding among feature label cells, and the strength of the connection between feature label cells and stimulus identity cells (which could be variable for variable numbers of features per stimulus; see STAR Methods). A high threshold for feature label cell firing means fewer co-active feature label cells at a given time (and hence fewer competing hypotheses), with the consequence that the leading feature label cell accounts for a higher fraction of overall firing after the softmax operation, leading to fewer saccades on average. Scaling down the strengths (i.e., gain) of connections from feature label cells to stimulus identity neurons increases the average number of saccades.

The activity of grid cells along each saccade vector (or just at the start and end locations) can be recorded and binned

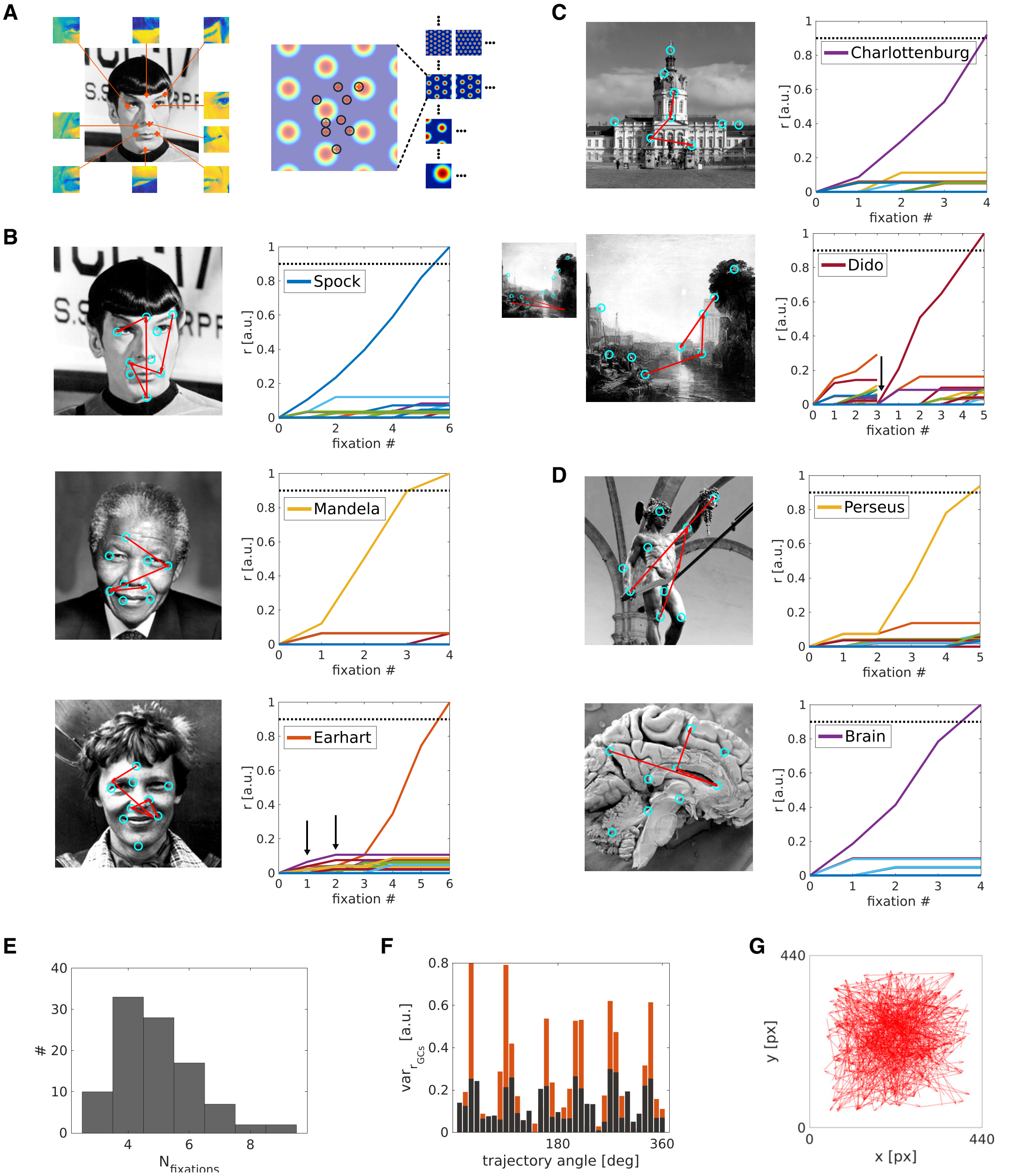

Figure 3. Recognition of Stimuli

(A) Salient features are associated with locations in the visual field via grid cells, and each location (red crosses in black circles) is encoded by the phases across the entire grid cell ensemble (4 out of 9 scales shown) as a population vector (i.e., a given pixel value across all grid cell rate maps).

(B) Saccade sequences (red arrows) superimposed on face stimuli (left). Cyan circles indicate the centers of all encoded local features. With each sampled feature, a firing rate of the corresponding identity cell is incremented (right). The dashed line indicates the decision threshold. Note, in panel 3, an initially wrong hypothesis (black arrows) is overtaken by the correct one.

(C) Saccades superimposed on scene stimuli. In panel 2, an initially wrong hypothesis yields misdirected saccades (relative to the true stimulus), which leads to an early reset (black arrow) because the predicted and actual outcome of the feature discrimination differ persistently. Starting from different initial features eventually leads to recognition.

(D) Recognition of object stimuli.

(E) Histogram of the number of saccades necessary for recognition across all stimuli.

(F) Variance of the activity across grid cells along each saccade vector (red bars) or just at the start and end locations (dark gray bars), binned according to the direction of the saccade (10-degree bins). 6-fold symmetry akin to fMRI data arises from the underlying symmetry of grid cells.

(G) All saccade vectors used for the analysis in (F). Image credit: Mr. Spock, Amelia Earhart, Dido Building Carthage: public domain images; Nelson Mandela, Schloss Charlottenburg, Brain, Perseus: Creative Commons Attribution.

according to the direction of the saccade. Plotting the firing rate variance across cells (normalized and baseline corrected, using 10° bins; Figure 3F) reveals a 6-fold symmetry (a few cells fire a lot if saccades align to grid axes, whereas many cells fire at a lower rate if they misalign), a direct consequence of the use of grid cells. Hence, the model is consistent with 6-fold modulation of the fMRI signal by eye-movement direction [18–20] if generation of the signal includes history-dependent factors such as seen in ''fMRI adaptation'' [32, 33]. The present model thus suggests a functional explanation for the 6-fold symmetry in fMRI signal amplitude in visual experimental paradigms, following those seen during navigation in virtual and cognitive spaces [34–36]. Figure 3G shows all saccade vectors used for the analysis.

Further advantages of coupling internally generated hypotheses to eye-movement vectors via grid cells become apparent when partially occluded stimuli are considered. The model is able to generate a meaningful succession of eye movements, even if the stimulus is partially occluded. Two types of simulations were conducted. In the first simulation, occlusions are modeled as regions of random intensity that weakly and indiscriminately drive feature label cells. In the second simulation, real-world occlusions were used. We randomly selected the occlusion from a set of 33 stimuli that consisted of approximately equal proportions of faces, scenes, objects, and generic textures (e.g., a brick wall, tree bark, etc.). Figure 4 shows examples of successful recognition of partially occluded stimuli. Starting with an un-occluded feature, the model generates saccades. If a saccade lands on the occlusion, the output of feature label cells is often weak and/or noisy (due to partial matches with many features) and does not necessarily pass the first threshold, which would allow it to contribute toward evidence accumulation (firing 2.8 SD above the mean, see Model Description), so the firing of the associated identity neuron does not increase (see step patterns in Figure 4). Thus, when the next saccade is performed, it is based on the currently most active stimulus identity cell (which is likely the previously most active one) without refinement of the hypothesis. The next target is simply selected by the return projection from the identity cell to its associated feature label cells, and the starting point of the saccade is given by current eye position (given by eye-motion updating of grid cells). Thus, saccades to and from expected but occluded features can still be performed. Moreover, the system can make use of the encoded stimulus layout by visiting non-occluded features from different (occluded or un-occluded) starting locations, though resets may be triggered more often, reflected in an increase number of total fixations (Figure 4G). With white noise occlusions, 97 out 99 stimuli were recognized. This number dropped to 86 with real-world occlusions. However, restricting the number of consecutive fixations on the occlusion to 1 improves performance to 92 recognized stimuli (see Figure 5G for summary statistics). We hypothesize that saccades to occlusions should be rare because they cannot be expected to contribute to evidence accumulation. In fact, such saccades may occur only if subjects are explicitly instructed to infer the location of hidden features. To the best of our knowledge, this ability has not been tested in the literature, although memory-guided saccades do provide indirect evidence. Lucas et al. [14] show that subjects can correctly place features (previously encountered as an array) on an empty screen, accompanied by fixations of the target areas; i.e., the locations of the features are somehow inferred. Importantly, re-running the same simulations with a new random seed does not lead to failed recognition in the same stimuli. In other words, a recognition failure is specific to a given order of saccade targets, and most possible orders of saccades produces correct recognition.

As outlined in the model overview, size invariance can be achieved by allowing a variable gain factor relating the magnitude of grid cell displacement vectors and the magnitude of oculo-motor output. Correctly estimating this gain depends on a size estimate of the stimulus relative to some memorized baseline value. This allows the model to recognize a previously encoded stimulus at different distances (i.e., a downscaled image; Figure 5A). In analogy to the effect of environmental change on grid cells in spatial memory [30], the first (foveal) sensory input is assumed to align the grid cell ensemble (accounting for positional variation). Between full-size and half-size versions of the same stimulus, no changes to the vector computation performed by the grid cell ensemble or the distance cell model are needed. Only the extent of the foveal array is downscaled to match the smaller image (e.g., corresponding to an attentional modulation determining the extent of the retinal image used). Simply scaling the gain of the magnitude of all saccade vectors by the same amount suffices to generate a successful sequence of saccades (Figures 5A and 5B). 98 out 99 stimuli were recognized when downscaled (Figure 5G), with a similar median number of fixations (Figure 5B) compared to default size stimuli.

Finally, disconnecting the grid system from the rest of the model leads to a sharp drop in recognition performance. We conducted two simulations, assuming that in the absence of grid cell-guided saccades bottom-up attention will select fixation targets randomly among all available targets. As a consequence, sensory predictions rarely match the outcome of feature discriminations, leading to poor evidence accumulation and/or a sharp increase in the number of resets. We define 10 resets without recognition as a failure to recognize the stimulus. Under these conditions, recognition performance drops to 40 recognized stimuli. Since it is unlikely that all potential targets for bottom-up attention are memorized, we conducted a second simulation where we included 5 additional targets that can attract attention (i.e., distractors) in each image. Attention can then select the next fixation target among 14 targets (9 memorized + 5 distractors). This reduces recognition performance further to 16 out of 99 items. Thus, lesions to the grid system may produce prosopagnosia-like symptoms (see Discussion).

DISCUSSION

We have presented a simple model of how the brain may calculate saccade vectors during recognition memory. The model is the first to suggest a specific role for grid cells in visual recognition and consistent with recent evidence of grid cells driven by eye movements [17–20]. The core predictions are that visual recognition memory will engage grid cells whenever the relative layout of multiple features must be taken into account, that lesions to the grid cell system will preclude this relational (i.e., configural) processing, and that the system allows an agent to infer

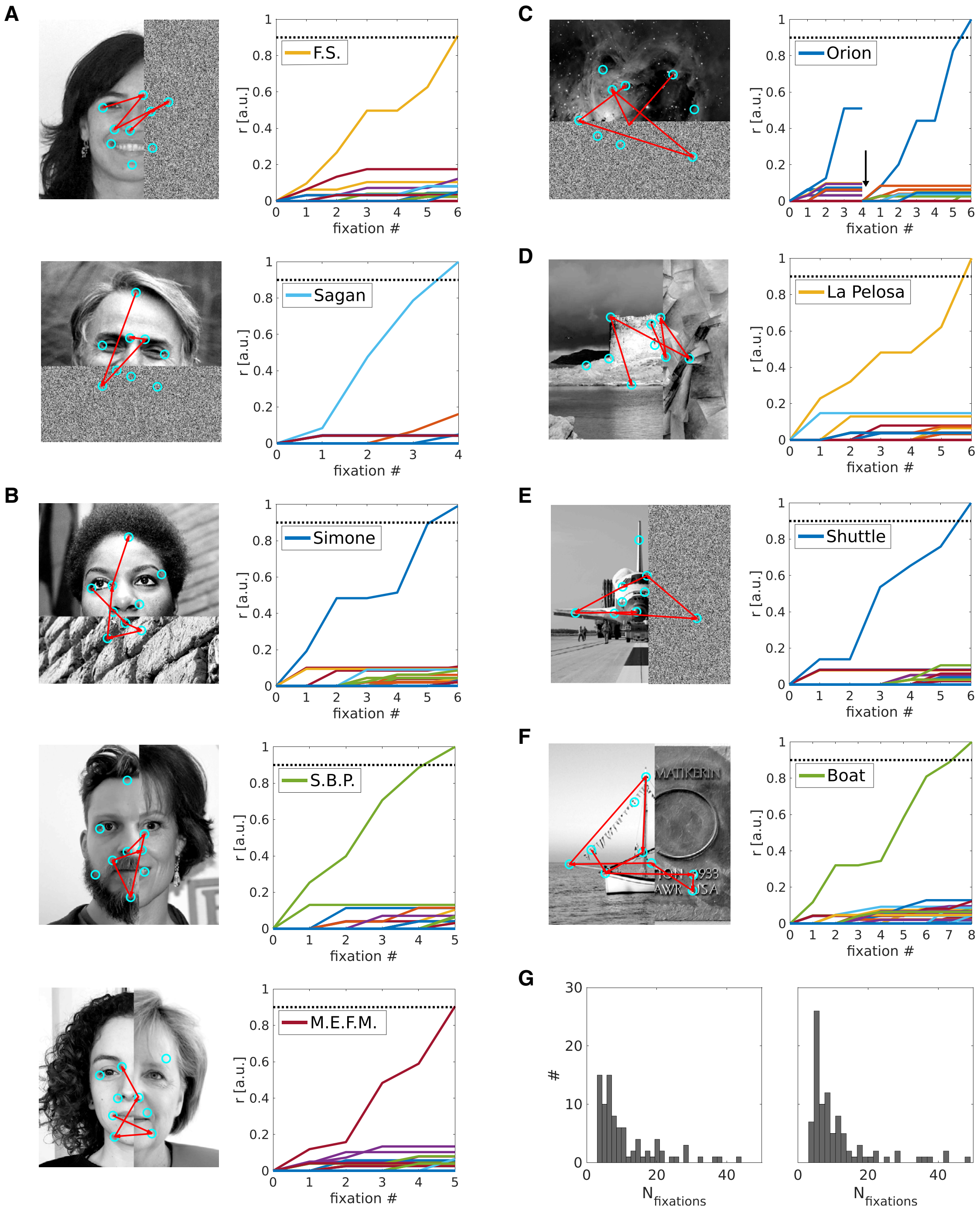

Figure 4. Occlusions

The (expected) locations of occluded features can still serve as start and end points of saccades, however, occluded features rarely increment the output of the associated identity cells (stair patterns in line plots).

(A) Face stimuli with white noise occlusions. Saccades sequences (red arrows) superimposed on face stimuli (left). Cyan circles indicate the centers of all encoded local features. With each sampled feature, a firing rate of the corresponding identity cell is incremented (right).

(B) Same as (A) with real-world occlusions.

(C and D) Scene stimuli with white noise (C) and real-world (D) occlusions.

(E and F) Object stimuli with white noise (E) and real-world (F) occlusions.

(G) The total number of fixations (including resets) for white noise (left) and real-world occlusions.

Image credit: F.S., S.B.P., M.E.F.M.: used with permission; Boat, La Pelosa Beach: supplied by author, with permission; Shuttle: public domain image; Carl Sagan, Nina Simone, Orion, Sigourney Weaver, Angela Merkel, brick wall, Picasso, Emmy Noether plaque: Creative Commons Attribution.

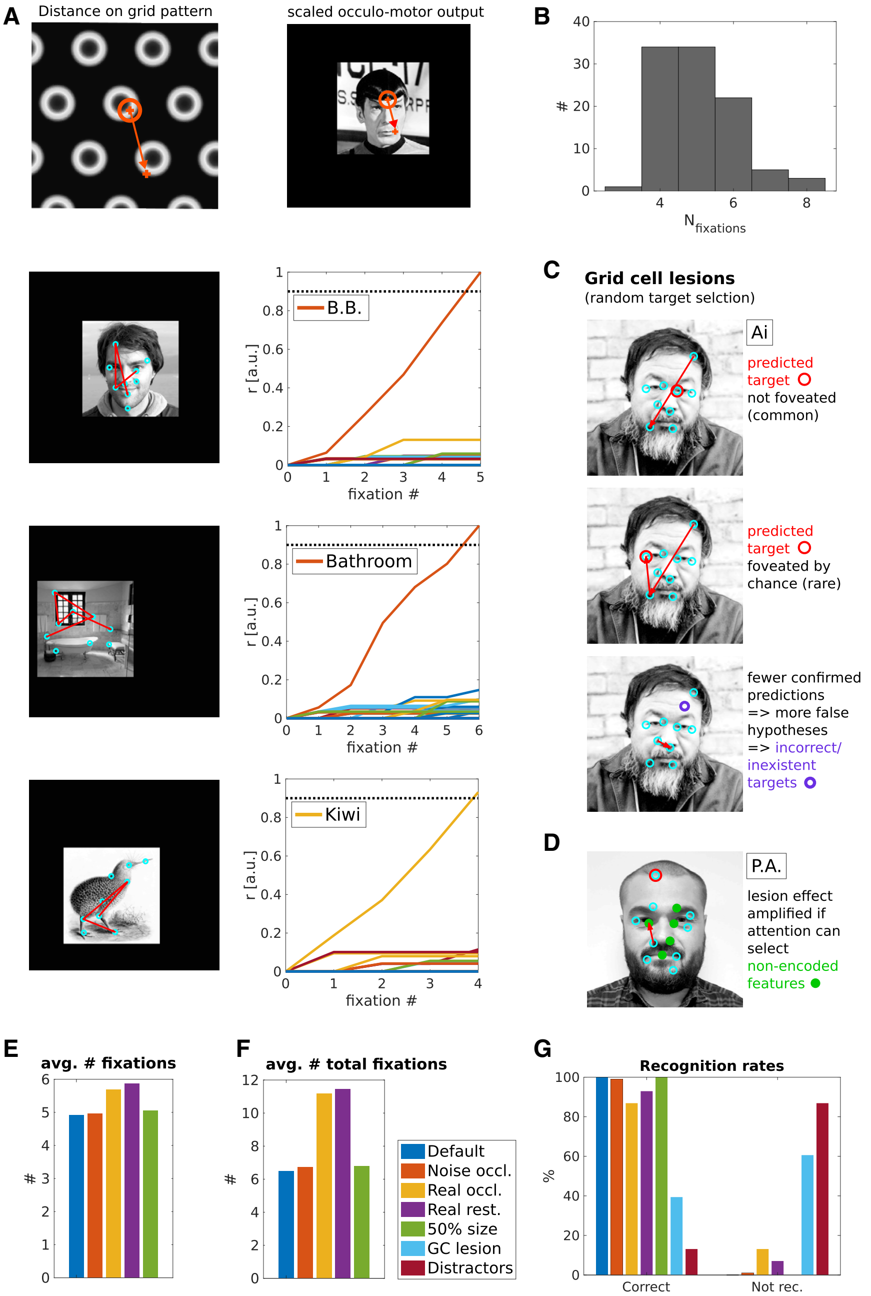

Figure 5. Size Invariance and Grid Cell Lesions

(A) Top: Illustration of the relationship between distances in "neural space" (on grid patterns) and visual space. Panels 2–4: Scaled down (50%) stimuli within the presentation frame of default size. Scaling the gain between the displacement vector on the grid pattern and the oculo-motor output uniformly for all saccades allows the model to sample all features of downscaled stimuli, leading to successful recognition irrespective of size. Saccade sequences (red arrows) are superimposed on stimuli (left). Cyan circles indicate the centers of all encoded local features. With each sampled feature, a firing rate of the corresponding identity cell is incremented (right).

(B) Histogram of the number of saccades (from last reset) necessary for recognition across all stimuli.

(C) Upon disconnecting grid cells from the model, it is assumed bottom-up attention will select among possible targets randomly. Panel 1: The next predicted feature (red circle) and the foveated feature rarely match, leading to poor evidence accumulation. Panel 2: By chance the next predicted feature (red circle) can be selected for foveation. Panel 3: The absence of confirmatory predictions (and thus facilitation among feature label cells) increases the number of false hypotheses of stimulus identity, leading to predicted features that are not present in the stimulus being viewed (purple circle). The behavior in panels 1 and 3 reduces recognition performance, while that in panel 2 contributes to residual recognition ability.

(D) Cyan circles indicate memorized features. Adding additional potential targets for bottom-up attention (i.e., distractors, filled green circles) exacerbates the performance drop.

(E–G) The average number of fixations from the last reset across conditions (E). The average number of fixations including resets (F). Recognition and failure rates (no recognition within 10 resets, "not rec.") for all tested conditions (G). Default condition, blue; white noise and real-world occlusions, red and yellow, respectively; real-world occlussions limited to one consecutive saccade towards the occlusion, purple; 50% shrunken stimuli, green; grid cell lesion with and without distractors, light blue and dark red, respectively.

Image credit: B.B., P.A.: used with permission; Mr. Spock, Ai Wei Wei, Kiwi, bathroom: public domain images.

the locations of occluded features. The model suggests how one-shot learning of exemplars may occur (paralleling one-shot learning of episodic memories in the hippocampal formation). Following this analogy, if deliberate recall is viewed as a top-down reconstruction of a previously experienced sensory representation, then the model also shows how relational information (sensory information tied to an arrangement of locations) could be made available for reinstatement. The specific visual mechanism presented here may extend to object recognition in other modalities [37].

In viewing tasks where whole scenes are presented within a constant border [17, 20], grid cells appear to be anchored to the borders, as are spatial grids in rodents across multiple sessions of navigation within a constant enclosure. In these experiments, the offset of grid cells relative to the enclosure only shifts in extreme circumstances, in which place cells "remap," like moving the entire enclosure to a different experimental room [30]. We suggest that in the absence of environmental anchor points, grid-like firing patterns would be anchored to a salient foreground stimulus that has to be recognized (e.g., when recognizing illuminated faces or objects in darkness). A strong prediction is that the grid patterns shift with the to-be-recognized object when presented in a new location. Grid cells could be anchored to the sensory scene by connections from place cells in the spatial case [38] and similarly by connections from feature label cells during visual recognition.

The implementation of the model is deliberately simplistic so as to focus on the basic mechanism proposed. Grid cells are implemented as a look-up table (firing rate maps). The remaining components of the model are also fairly conventional (visual feature detectors, identity or concept cells). The model suggests a parsimonious encoding of relative locations. The distance cell and grid cell systems are not stimulus specific. The number of stimulus identity cells and feature label cells per stimulus is of the order of the number of identifying (high-level) features, though the model does not preclude that visual features could be also encoded in a distributed manner. Despite this simplicity, tentative anatomical links can be established. The stimulus identity cells in the model are reminiscent of concept cells found in the human hippocampus [39]. The use of hippocampal cells in conjunction with entorhinal grid cells would be consistent with the involvement of the hippocampal formation in some perceptual tasks [14, 40, 41]. It may also account for medial temporal lobe interactions with the visual system during visual recognition of relational information and object-location binding [22–24], and for memory-guided attention [13, 14], as well as during navigation [42, 43].

The hippocampus and the parahippocampal gyrus also appear to support retrieval of pre-experimental knowledge about stimulus location within a scene [13] or pre-experimental knowledge about specific faces [44]. The fact that the hippocampus is required to recognize familiar faces, but not new, recently seen faces [45], suggests that the grid cell system may be predominantly required to support long-term relational memory for familiar identifiable stimuli.

The presence of object, scene, and face-specific processing streams in anterior temporal lobe suggests that recognition memory for all these stimulus categories could benefit from anatomically close grid cell representations in entorhinal cortex. Intriguingly, face processing in Macaques engages entorhinal cortex in addition to homologs of human face-processing areas [46]. The prominent role of perirhinal cortex in the processing and memory of objects and faces [47, 48] suggests that neural populations akin to feature label cells may reside there. Face-selective patches have also been identified in the ventral anterior temporal lobes, adjacent to perirhinal and entorhinal cortices [49, 50]. Similarly, the anatomical adjacency of the parahippocampal areas to entorhinal cortex makes it a candidate structure for cells that may represent salient parts of a scene, similar to feature label cells in the model. Supporting this view, anatomical projections have been reported between entorhinal cortex and parahippocampal areas TH and TF, as well as IT/TE and perirhinal cortex [51–53]. Intriguingly, Blatt et al. [54] report that these same areas (TF, TE) are connected to LIP, a structure crucial for saccade execution, which is directly connected to the superior colliculus (SC) and the frontal eye fields (FEFs). It has been suggested that memory-guided saccades rely on SC being subject to top-down influence via FEF and LIP [55]. A saccade could then be executed to the location in the visual field indicated by the SC, which most closely matches the endpoint of a grid cell-derived saccade vector [25]. Grid cell-based computations in and near entorhinal cortex for memory-guided saccades should then precede motor output and thus corollary discharge mediated by thalamic pathways involving the SC (for review, see [56, 57],).

We note that categorical object recognition (e.g., distinguishing a car from an elephant) need not make use of the proposed mechanism (the different categories can be distinguished by their constituent features irrespective of their layout). However, when fine within-category judgments of feature layout are required, relational processing may be necessary, for which grid cells can provide the neural substrate. Focal lesions to grid cell systems should then disrupt relational memory processing (with bottom-up attentional saccade guidance preserved), yielding deficits in the recognition of exemplars with spared category recognition. Interestingly, Damasio et al. [58] report that true prosopagnosics are still able to recognize a face as a face and name it as such (when the query is presented similarly to objects) but are unable to report specific identity. Additionally, recognizing object exemplars can also be impaired (e.g., a bird watcher being unable to recognize individual birds [58]). We have shown that prosopagnosia-like symptoms may arise from disconnecting grid cells (and/or stimulus identity cells) from neurons (akin to feature label cells in the model) in upstream areas. Although sequences of saccades can in principle be solely driven by bottom-up attention to salient features in the visual field, the relational content is essential for deliberate recall. Further support comes from experiments showing that recognition of multi-featured items is more difficult when stimuli are scrambled [12, 59]. Moreover, bottom-up attention alone cannot account for eye movements during acts of visual imagery [60, 61], i.e., when specific eye-movement patterns are induced from memory [21, 62, 63].

On a purely behavioral level, an interesting prediction is that idiosyncratic differences between subjects could transfer between apparently radically different tasks, if they employ grid cells. For example, bad navigators could also be bad at recognizing exemplars, with the strongest effect likely occurring for familiar, non-famous faces and difficulty-matched groups of object or scene exemplars [64]. Although configural processing has mainly been associated with faces [65], studies employing ''greebles'' show that the hallmarks of holistic, face-like processing can be observed for objects, potentially related to expertise in distinguishing individual (object) exemplars [66, 67].

Since successful saccades in the model depend on the relative arrangement of features, the model is compatible with the notion of holistic processing, as exemplified by the ''composite face effect'' [68], the ''part-whole recognition effect'' [12], and the difficulty to process and recognize upside-down faces [69], for which saccades would be guided in the wrong direction. Notably, Tanaka and Sengco [12] have shown that displacement of one facial feature (e.g., increasing eye separation) reduces the recognition rate for other features within the same face, a finding that maps well onto the present account in which individual features determine the next saccade vector. The successful recognition of stimuli that are partially occluded is also a consequence of the vector-based, relational mechanism and may form the basis of our ability to infer the locations of occluded features.

If size invariance is accomplished within the grid cell system, then the present account predicts a rescaling of visual grids with the size of the stimulus in the visual field (cf. [31].). Regarding rotations, classic models of object recognition have proposed that either viewpoint-invariant (3D) representations of objects are stored [70], or that multiple canonical views of an object

are stored, with intermediate views synthesized by interpolation [71, 72]. It has been suggested [73] that both view-invariant (structural) as well as view-based approaches are implemented in the brain, and that structural-descriptions might support category level classification, whereas view-based mechanisms could support item-specific recognition. The mechanism we have proposed here could, for instance, operate on one or several individual view-based, canonical representations (possibly with separate feature label cells for each view, but the same identity neuron(s) across views) in anterior temporal regions, corresponding to different rotation angles, and interpolate saccade length between the two closest views. Attentional mechanisms could facilitate small corrective saccades (i.e., microsaccades) if the calculated saccade lands sufficiently close to the target.

Finally, the present account can accommodate a broader Bayesian interpretation of perception. For instance, information about context could be integrated (as prior beliefs), similar to the facilitatory bias of predictions in the current model, applied to a subset of stimuli one is expecting to encounter. For example, at a workplace one would expect to encounter colleagues and hence firing of cells representing their features and identities may be preferentially incremented. However, note that individual memory-dependent saccades must be guided by one and only one hypothesis at a time in order to move the fovea to the location of an expected feature, rather than, e.g., between two competing locations. Also note, already the first firing of an identity cell represents the formation of a hypothesis or belief. Integrated with contextual and gist information, a very small number of saccades may suffice to reach sufficient confidence.

Conclusions

We have presented a mechanistic model of visual recognition memory via grid cells, better known for their role in spatial navigation. However, grid-like activity in visual paradigms suggests that the same neural circuit could also contribute to visual processing. Vestibular and bodily motor efference signals could drive grid cells during path integration and large-scale spatial navigation, and occulomotor inputs could update the same cells when an agent is engaged in a visual discrimination task. Similarly, by extension grid cells could provide a compact code for locations in any continuous space, e.g., in conceptual [36] or auditory [74] spaces. The present model offers an explanation as to why medial temporal structures are sometimes involved in recognition memory and supports the emerging notion that grid cells are part of a universal representational system, where the inputs determine the exact response properties of grid cells to amend their neural code to a wide range of tasks.

STAR★METHODS

Detailed methods are provided in the online version of this paper and include the following:

- KEY RESOURCE TABLE
- CONTACT FOR REAGENT AND RESOURCE SHARING
- EXPERIMENTAL MODEL AND SUBJECT DETAILS
- METHOD DETAILS
  - Model overview
  - Action/Perception cycles
  - Sensory predictions and resets
  - Grid Cells and Vector Computations
  - Training phase
  - Feature detection and ambiguity
  - Position Invariance
  - Size Invariance
- QUANTIFICATION AND STATISTICAL ANALYSIS
- DATA AND SOFTWARE AVAILABILITY

SUPPLEMENTAL INFORMATION

Supplemental Information includes one figure and two tables and can be found with this article online at https://doi.org/10.1016/j.cub.2019.01.077.

ACKNOWLEDGMENTS

We acknowledge funding by the ERC Advanced grant NEUROMEM, Wellcome, and the European Union's Horizon 2020 research and innovation programme under grant agreement no. 785907 Human Brain Project SGA2. We thank Daniel Bush, James Bisby, Sofie Meyer, Alexandra Constantinescu, Jesse Geerts, and Talfan Evans for comments and useful discussions.

AUTHOR CONTRIBUTIONS

A.B. conceptualized, designed, and implemented the model in code, created visualizations, and wrote the paper. N.B conceptualized the model, wrote the paper, and secured funding.

DECLARATION OF INTERESTS

The authors declare no competing interests.

Received: September 28, 2018
Revised: December 23, 2018
Accepted: January 30, 2019
Published: March 7, 2019

REFERENCES

1. Fukushima, K., and Miyake, S. (1982). Neocognitron: A new algorithm for pattern recognition tolerant of deformations and shifts in position. Pattern Recognit. *15*, 455–469.
2. Riesenhuber, M., and Poggio, T. (1999). Hierarchical models of object recognition in cortex. Nat. Neurosci. *2*, 1019–1025.
3. LeCun, Y., Bengio, Y., and Hinton, G. (2015). Deep learning. Nature *521*, 436–444.
4. Hubel, D.H., and Wiesel, T.N. (1962). Receptive fields, binocular interaction and functional architecture in the cat's visual cortex. J. Physiol. *1*, 106–154.
5. Yarbus, A.L. (1967). Eye Movements During Perception of Complex Objects (Springer).
6. Neisser, U. (1967). Cognitive Psychology. (Appleton-Century-Crofts).
7. Noton, D., and Stark, D. (1970). Scanpaths in eye movements during Paftern perception. Science *171*, 308–311.
8. Gregory, R.L. (1980). Perceptions as hypotheses. Philos. Trans. R. Soc. Lond. B Biol. Sci. *290*, 181–197.
9. Friston, K., Adams, R.A., Perrinet, L., and Breakspear, M. (2012). Perceptions as hypotheses: Saccades as experiments. Front. Psychol. *28*, 151.
10. Gilchrist, I.D., Brown, V., and Findlay, J.M. (1997). Saccades without eye movements. Nature *390*, 130–131.
11. Henderson, J.M., Williams, C.C., and Falk, R.J. (2005). Eye movements are functional during face learning. Mem. Cognit. *33*, 98–106.

12. Tanaka, J.W., and Sengco, J.A. (1997). Features and their configuration in face recognition. Mem. Cognit. *25*, 583–592.
13. Summerfield, J.J., Lepsien, J., Gitelman, D.R., Mesulam, M.M., and Nobre, A.C. (2006). Orienting attention based on long-term memory experience. Neuron *49*, 905–916.
14. Lucas, H.D., Duff, M.C., and Cohen, N.J. (2018). The hippocampus promotes effective saccadic information gathering in humans. J. Cogn. Neurosci. *2018*, 1–6.
15. O'Keefe, J., and Conway, D.H. (1978). Hippocampal place units in the freely moving rat: Why they fire where they fire. Exp. Brain Res. *31*, 573–590.
16. Hafting, T., Fyhn, M., Molden, S., Moser, M.B., and Moser, E.I. (2005). Microstructure of a spatial map in the entorhinal cortex. Nature *436*, 801–806.
17. Killian, N.J., Jutras, M.J., and Buffalo, E.A. (2012). A map of visual space in the primate entorhinal cortex. Nature *491*, 761–764.
18. Nau, M., Navarro Schröder, T., Bellmund, J.L.S., and Doeller, C.F. (2018). Hexadirectional coding of visual space in human entorhinal cortex. Nat. Neurosci. *21*, 188–190.
19. Nau, M., Julian, J.B., and Doeller, C.F. (2018). How the brain's navigation system shapes our visual experience. Trends Cogn. Sci. *22*, 810–825.
20. Julian, J.B., Keinath, A.T., Frazzetta, G., and Epstein, R.A. (2018). Human entorhinal cortex represents visual space using a boundary-anchored grid. Nat. Neurosci. *21*, 191–194.
21. Hannula, D.E., Ryan, J.D., Tranel, D., and Cohen, N.J. (2007). Rapid onset relational memory effects are evident in eye movement behavior, but not in hippocampal amnesia. J. Cogn. Neurosci. *19*, 1690–1705.
22. Olsen, R.K., Lee, Y., Kube, J., Rosenbaum, R.S., Grady, C.L., Moscovitch, M., and Ryan, J.D. (2015). The role of relational binding in item memory: Evidence from face recognition in a case of developmental amnesia. J. Neurosci. *35*, 5342–5350.
23. Eichenbaum, H., Yonelinas, A.P., and Ranganath, C. (2007). The medial temporal lobe and recognition memory. Annu. Rev. Neurosci. *30*, 123–152.
24. Pertzov, Y., Miller, T.D., Gorgoraptis, N., Caine, D., Schott, J.M., Butler, C., and Husain, M. (2013). Binding deficits in memory following medial temporal lobe damage in patients with voltage-gated potassium channel complex antibody-associated limbic encephalitis. Brain *136*, 2474–2485.
25. Killian, N.J., Potter, S.M., and Buffalo, E.A. (2015). Saccade direction encoding in the primate entorhinal cortex during visual exploration. Proc. Natl. Acad. Sci. USA *112*, 15743–15748.
26. McNaughton, B.L., Battaglia, F.P., Jensen, O., Moser, E.I., and Moser, M.B. (2006). Path integration and the neural basis of the 'cognitive map'. Nat. Rev. Neurosci. *7*, 663–678.
27. Kubie, J.L., and Fenton, A.A. (2012). Linear look-ahead in conjunctive cells: An entorhinal mechanism for vector-based navigation. Front. Neural Circuits. *6*, 20.
28. Erdem, U.M., and Hasselmo, M. (2012). A goal-directed spatial navigation model using forward trajectory planning based on grid cells. Eur. J. Neurosci. *35*, 916–931.
29. Bush, D., Barry, C., Manson, D., Burgess, N., Barry, C., Manson, D., and Burgess, N. (2015). Using grid cells for navigation. Neuron *87*, 507–520.
30. Fyhn, M., Hafting, T., Treves, A., Moser, M.-B., and Moser, E.I. (2007). Hippocampal remapping and grid realignment in entorhinal cortex. Nature *446*, 190–194.
31. Barry, C., Hayman, R., Burgess, N., and Jeffery, K.J. (2007). Experience-dependent rescaling of entorhinal grids. Nat. Neurosci. *10*, 682–684.
32. Grill-Spector, K., and Malach, R. (2001). fMR-adaptation: A tool for studying the functional properties of human cortical neurons. Acta Psychol. (Amst.) *107*, 293–321.
33. Doeller, C.F., Barry, C., and Burgess, N. (2010). Evidence for grid cells in a human memory network. Nature *463*, 657–661.
34. Bellmund, J.L., Deuker, L., Navarro Schröder, T., and Doeller, C.F. (2016). Grid-cell representations in mental simulation. Elife *30*, e17089.
35. Horner, A.J., Bisby, J.A., Zotow, E., Bush, D., and Burgess, N. (2016). Grid-like processing of imagined navigation. Curr. Biol. *21*, 842–847.
36. Constantinescu, A.O., O'Reilly, J.X., and Behrens, T.E.J. (2016). Organizing conceptual knowledge in humans with a gridlike code. Science *352*, 1464–1468.
37. Hawkins, J., Lewis, M., Klukas, M., Purdy, S., and Ahmad, S. (2019). A framework for intelligence and cortical function based on grid cells in the neocortex. Front. Neural Circuits *12*, 121.
38. O'Keefe, J., and Burgess, N. (2005). Dual phase and rate coding in hippocampal place cells: Theoretical significance and relationship to entorhinal grid cells. Hippocampus *15*, 853–866.
39. Quiroga, R.Q. (2012). Concept cells: The building blocks of declarative memory functions. Nat. Rev. Neurosci. *13*, 587–597.
40. Lee, A.C.H., Buckley, M.J., Pegman, S.J., Spiers, H., Scahill, V.L., Gaffan, D., Bussey, T.J., Davies, R.R., Kapur, N., Hodges, J.R., and Graham, K.S. (2005). Specialization in the medial temporal lobe for processing of objects and scenes. Hippocampus *15*, 782–797.
41. Barense, M.D., Henson, R.N.A., and Graham, K.S. (2011). Perception and conception: Temporal lobe activity during complex discriminations of familiar and novel faces and objects. J. Cogn. Neurosci. *23*, 3052–3067.
42. Sherrill, K.R., Chrastil, E.R., Ross, R.S., Erdem, U.M., Hasselmo, M.E., and Stern, C.E. (2015). Functional connections between optic flow areas and navigationally responsive brain regions during goal-directed navigation. Neuroimage *118*, 386–396.
43. Saleem, A.B., Diamanti, E.M., Fournier, J., Harris, K.D., and Carandini, M. (2018). Coherent encoding of subjective spatial position in visual cortex and hippocampus. Nature *562*, 124–127.
44. Trinkler, I., King, J.A., Doeller, C.F., Rugg, M.D., and Burgess, N. (2009). Neural bases of autobiographical support for episodic recollection of faces. Hippocampus *19*, 718–730.
45. Bird, C.M., and Burgess, N. (2008). The hippocampus supports recognition memory for familiar words but not unfamiliar faces. Curr. Biol. *18*, 1932–1936.
46. Ku, S.P., Tolias, A.S., Logothetis, N.K., and Goense, J. (2011). fMRI of the face-processing network in the ventral temporal lobe of awake and anesthetized macaques. Neuron *70*, 352–362.
47. Brown, M.W., and Aggleton, J.P. (2001). Recognition memory: What are the roles of the perirhinal cortex and hippocampus? Nat. Rev. Neurosci. *2*, 51–61.
48. Murray, E.A., Bussey, T.J., and Saksida, L.M. (2007). Visual perception and memory: A new view of medial temporal lobe function in primates and rodents. Annu. Rev. Neurosci. *30*, 99–122.
49. Jonas, J., Jacques, C., Liu-Shuang, J., Brissart, H., Colnat-Coulbois, S., Maillard, L., and Rossion, B. (2016). A face-selective ventral occipito-temporal map of the human brain with intracerebral potentials. Proc. Natl. Acad. Sci. USA *113*, E4088–E4097.
50. Collins, J.A., and Olson, I.R. (2014). Beyond the FFA: The role of the ventral anterior temporal lobes in face processing. Neuropsychologia *61*, 65–79.
51. Kosel, K.C., Van Hoesen, G.W., and Rosene, D.L. (1982). Non-hippocampal cortical projections from the entorhinal cortex in the rat and rhesus monkey. Brain Res. *244*, 201–213.
52. Insausti, R., Herrero, M.T., and Witter, M.P. (1997). Entorhinal cortex of the rat: Cytoarchitectonic subdivisions and the origin and distribution of cortical efferents. Hippocampus *7*, 146–183.
53. Saleem, K.S., and Tanaka, K. (1996). Divergent projections from the anterior inferotemporal area TE to the perirhinal and entorhinal cortices in the macaque monkey. J. Neurosci. *16*, 4757–4775.
54. Blatt, G.J., Andersen, R.A., and Stoner, G.R. (1990). Visual receptive field organization and cortico-cortical connections of the lateral intraparietal area (area LIP) in the macaque. J. Comp. Neurol. *299*, 421–445.

55. Pierrot-Deseilligny, C., Rivaud, S., Gaymard, B., and Agid, Y. (1991). Cortical control of memory-guided saccades in man. Exp. Brain Res. *83*, 607–617.
56. Wurtz, R.H., McAlonan, K., Cavanaugh, J., and Berman, R.A. (2011). Thalamic pathways for active vision. Trends Cogn. Sci. *15*, 177–184.
57. Sommer, M.A., and Wurtz, R.H. (2008). Brain circuits for the internal monitoring of movements. Annu. Rev. Neurosci. *31*, 317–338.
58. Damasio, A.R., Damasio, H., and Van Hoesen, G.W. (1982). Prosopagnosia: Anatomic basis and behavioral mechanisms. Neurology *32*, 331–341.
59. Collishaw, S.M., and Hole, G.J. (2000). Featural and configurational processes in the recognition of faces of different familiarity. Perception *29*, 893–909.
60. Brandt, S.A., and Stark, L.W. (1997). Spontaneous eye movements during visual imagery reflect the content of the visual scene. J. Cogn. Neurosci. *9*, 27–38.
61. Bone, M.B., St-Laurent, M., Dang, C., McQuiggan, D.A., Ryan, J.D., and Buchsbaum, B.R. (2018). Eye movement reinstatement and neural reactivation during mental imagery. Cereb. Cortex. Published online February 3, 2018. https://doi.org/10.1093/cercor/bhy014.
62. Meister, M.L.R., and Buffalo, E.A. (2016). Getting directions from the hippocampus: The neural connection between looking and memory. Neurobiol. Learn. Mem. *134* (Pt A), 135–144.
63. Urgolites, Z.J., Smith, C.N., and Squire, L.R. (2018). Eye movements support the link between conscious memory and medial temporal lobe function. Proc. Natl. Acad. Sci. USA *115*, 7599–7604.
64. Zachariou, V., Safiullah, Z.N., and Ungerleider, L.G. (2017). The fusiform and occipital face areas can process a nonface category equivalently to faces. J. Cogn. Neurosci. *30*, 1–8.
65. Maurer, D., Grand, R.L., and Mondloch, C.J. (2002). The many faces of configural processing. Trends Cogn. Sci. *6*, 255–260.
66. Gauthier, I., Tarr, M.J., Anderson, A.W., Skudlarski, P., and Gore, J.C. (1999). Activation of the middle fusiform 'face area' increases with expertise in recognizing novel objects. Nat. Neurosci. *2*, 568–573.
67. Campbell, A., and Tanaka, J.W. (2018). Inversion impairs expert budgerigar identity recognition: A face-like effect for a nonface object of expertise. Perception *47*, 647–659.
68. Young, A.W., Hellawell, D., and Hay, D.C. (2013). Configurational information in face perception. Perception *42*, 1166–1178.
69. Yin, R.K. (1969). Looking at upide-down faces. J. Exp. Psychol. *81*, 141–145.
70. Marr, D., and Nishihara, H.K. (1978). Representation and recognition of the spatial organization of three-dimensional shapes. Proc. R. Soc. Lond. B Biol. Sci. *200*, 269–294.
71. Logothetis, N.K., Pauls, J., Bülthoff, H.H., and Poggio, T. (1994). View-dependent object recognition by monkeys. Curr. Biol. *4*, 401–414.
72. Blanz, V., Tarr, M.J., and Bülthoff, H.H. (1999). What object attributes determine canonical views? Perception *28*, 575–599.
73. Tarr, M.J., and Bülthoff, H.H. (1998). Image-based object recognition in man, monkey and machine. Cognition *67*, 1–20.
74. Aronov, D., Nevers, R., and Tank, D.W. (2017). Mapping of a non-spatial dimension by the hippocampal-entorhinal circuit. Nature *543*, 719–722.
75. Bush, D., and Burgess, N. (2014). A hybrid oscillatory interference/continuous attractor network model of grid cell firing. J. Neurosci. *34*, 5065–5079.
76. Burak, Y., and Fiete, I.R. (2009). Accurate path integration in continuous attractor network models of grid cells. PLoS Comput. Biol. *5*, e1000291.
77. Hasselmo, M.E., Rolls, E.T., and Baylis, G.C. (1989). The role of expression and identity in the face-selective responses of neurons in the temporal visual cortex of the monkey. Behav. Brain Res. *32*, 203–218.
78. Perrett, D.I., Smith, P.A., Potter, D.D., Mistlin, A.J., Head, A.S., Milner, A.D., and Jeeves, M.A. (1984). Neurones responsive to faces in the temporal cortex: Studies of functional organization, sensitivity to identity and relation to perception. Hum. Neurobiol. *3*, 197–208.
79. Fiete, I.R., Burak, Y., and Brookings, T. (2008). What grid cells convey about rat location. J. Neurosci. *28*, 6858–6871.
80. Mathis, A., Herz, A.V.M., and Stemmler, M. (2012). Optimal population codes for space: Grid cells outperform place cells. Neural Comput. *24*, 2280–2317.
81. Stensola, H., Stensola, T., Solstad, T., Frøland, K., Moser, M.B., and Moser, E.I. (2012). The entorhinal grid map is discretized. Nature *492*, 72–78.
82. Chen, Q., and Verguts, T. (2010). Beyond the mental number line: A neural network model of number-space interactions. Cognit. Psychol. *60*, 218–240.
83. Poletti, M., and Rucci, M. (2016). A compact field guide to the study of microsaccades: Challenges and functions. Vision Res. *118*, 83–97.
84. Peel, T.R., Hafed, Z.M., Dash, S., Lomber, S.G., and Corneil, B.D. (2016). A causal role for the cortical frontal eye fields in microsaccade deployment. PLoS Biol. *14*, e1002531.
85. Willeke, K., Tian, X., Bellet, J., Ramirez-Cardenas, A., and Hafed, Z.M. (2017). Memory-guided microsaccades. bioRxiv. https://doi.org/10.1101/539205.
86. Kagan, I., and Hafed, Z.M. (2013). Active vision: Microsaccades direct the eye to where it matters most. Curr. Biol. *23*, R712–R714.
87. Wilming, N., König, P., König, S., and Buffalo, E.A. (2018). Entorhinal cortex receptive fields are modulated by spatial attention, even without movement. eLife *7*, e31745.
88. Baddeley, R.J., and Tatler, B.W. (2006). High frequency edges (but not contrast) predict where we fixate: A Bayesian system identification analysis. Vision Res. *46*, 2824–2833.
89. Samonds, J.M., Geisler, W.S., and Priebe, N.J. (2018). Natural image and receptive field statistics predict saccade sizes. Nat. Neurosci. *21*, 1591–1599.
90. Nadasdy, Z., Nguyen, T.P., Török, Á., Shen, J.Y., Briggs, D.E., Modur, P.N., and Buchanan, R.J. (2017). Context-dependent spatially periodic activity in the human entorhinal cortex. Proc. Natl. Acad. Sci. USA *114*, E3516–E3525.

STAR★METHODS

KEY RESOURCE TABLE

REAGENT or RESOURCE	SOURCE	IDENTIFYIER
Software and Algorithms		
MATLAB R2017b	https://www.mathworks.com/	https://www.mathworks.com/
Custom MATLAB Code	This Article	https://github.com/bicanski

CONTACT FOR REAGENT AND RESOURCE SHARING

Further information and requests for resources/code should be directed to the Lead Contact, Andrej Bicanski (andrej.bicanski@gmail.com).

EXPERIMENTAL MODEL AND SUBJECT DETAILS

Ten human subjects, personal acquaintances of the author (eight male and two female; ages 30–45) agreed to have their picture used in the study (as input to the model). Subjects provided informed written consent before donating a picture.

METHOD DETAILS

Model overview

All non-grid cells in the model are simple connectionist neurons with rectified linear output. Since grid cells exist as canonical firing rate maps (see below for details), their firing rates can be looked up as a function of eye position (for related models of grid cell firing dynamics see e.g., [75, 76]).

Grayscale images (440x440 pixels) represent pre-processed visual input to the model. In addition to the grid cell and distance cell components, the model consists of: a small square array of cells, representing pre-processed outputs from a simplified fovea (61x61 cells/pixels) with associated sensory cells (see next paragraph); a small number of 'feature label cells' (one for each salient feature within a familiar stimulus, here 9 per stimulus); and a single stimulus identity cell per image (representing the abstract identity of the stimulus, see e.g., [39, 77, 78]).

Banks of sensory neurons (playing the role of feature detectors, one bank associated with each foveal pixel) are implemented as cells with Gaussian tuning curves across possible (grayscale) pixel values. Individual cell exhibit a FWHM (full width at half maximum) of approximately 10% of the range of possible pixel values [0,255]. Thus, when a given input is presented by the fovea, sensory neurons express a characteristic response, indicative of the attended feature, though subject to noise (see below).

Stimuli (the grayscale images) are first presented in a training phase (see below for details). the model learns Hebbian associations between the following cell types. First, as an individual component feature (e.g., for the nose, eyes, lips etc. of a given face) of a stimulus is foveated it generates a characteristic response among the feature detectors. A Hebbian association between the array of feature detectors and an individual (newly recruited) feature label cell (representing the foveated component feature of a stimulus) is then learned. Second, a connection between feature label cells and the locations of those features in the visual field (represented by grid cell population vectors) is learned. Finally, all feature label cells belonging to a stimulus are associated – bi-directionally – to a stimulus identity cell, representing the abstract identity of a stimulus. That is, each stimulus identity cell receives connections from a small number of feature label cells representing the component features of that stimulus, and has a return projection to the same feature label cells (Figure 1E). Once the model has learned the necessary associations, its recognition memory is tested by presenting stimuli from the training set.

Action/Perception cycles

An action-perception cycle consists of the following steps: The foveal array is centered on a given feature (we assume that the first feature is selected by bottom-up attentional mechanisms, which are not modeled here). Feature detectors (perception) drive the feature label cells which (partially) match the attended feature. Feature label cells must exceed 2.8 standard deviations with respect to the firing rates of all feature label cells to be eligible to contribute evidence. After this thresholding a softmax operation is applied to ensure a sparse code among feature label cells. Active feature label cells drive their associated stimulus identity cells, generating competing hypotheses about the stimulus identity. The most active identity cell then determines the computation of the vector for the next saccade (action) in the following way. The current location of the fovea is represented by a population vector of grid cell activity, which is updated by eye-movements analogously to how grid cell firing is updated by self-motion during navigation [26, 76]. This yields the starting point of the saccade vector. Previously active feature label cells (including the one for the currently

foveated feature) are reset to zero and the most active stimulus identity cell (representing the leading hypothesis) randomly selects the next feature label cell via its return-projection (feature label cells representing already visited features are permanently inhibited). Randomness is given by weak noise on the back-projection and winner-take-all dynamics select the feature label cell, which is in turn associated with its own grid cell population vector (yielding the end point of the next saccade vector). Given the starting and end points of the next saccade, the distance cell system outputs the vector required to update foveal position (see below for vector computations), allowing the system to sample another portion of the visual field. The cycle then repeats while stimulus identity cells accumulate firing across cycles until a firing rate 'decision threshold' has been reached (at which point the leading hypothesis about the identity of the stimulus is accepted and identity cells reset to zero before the next stimulus is presented). The increment in firing of a stimulus identity cell (i.e., the gain on weights from feature label cells to stimulus identity cells; parameter *FLC2ID* in Table S2), and the decision threshold are free parameters. They could be adapted in situations where sensory input is more or less reliable, setting a lower recognition threshold (or a larger increment) would facilitate faster recognition, potentially at the expense of accuracy. It could also be a function of the number of available component features, thus accounting for variable numbers of available features between stimuli. If the decision threshold is not reached once all component features have been visited (which happens rarely), all permanently inhibited feature label cells (i.e., coding for already visited features) are disinhibited and the process continues.

Sensory predictions and resets

In addition to specifying the endpoint of the next saccade via associated grid cells, the feature label cell that has been selected by the return projection of the leading stimulus identity neuron also represents a prediction. Once the fovea relocates, and the next sensory discrimination is carried out, the maximally active feature label cell should be the predicted one. This prediction is incorporated as a facilitatory effect, boosting the firing of the predicted feature label cell in the next cycle by a factor (two), prior to the application of the softmax operation across all feature label cells. If the predicted feature label cell is not the most active one after the next sensory discrimination, a mismatch is registered. At the third mismatch event the system resets (i.e., the current hypotheses are all rejected), beginning with different component feature. This procedure allows for early rejection of false hypotheses, which will otherwise produce saccades that do not take the fovea to expected features. Figure S1 details the effect of sensory predictions. Note that multiple failures to reach the decision threshold could also be used to infer that the attended stimulus is unfamiliar.

Grid Cells and Vector Computations

Grid cells have been suggested to provide a spatial metric that supports path integration (by integrating self-motion inputs) and vector navigation [27–29]. The spatial periodicity of grid cells at different scales suggests that they provide a compact code for location, and that they can uniquely encode locations within a space much larger than the largest grid scale [29, 79, 80]. Grid cells are implemented as canonical firing rate maps which act as a look-up table. Each map consists of a matrix of the same dimensions as the PC sheet (440x440 pixels) and is computed as 60 degrees offset, superimposed cosine waves using the following set of equations.

$$b_0 = \begin{pmatrix} cos(0) \\ sin(0) \end{pmatrix} b_1 = \begin{pmatrix} cos\left(\frac{\pi}{3}\right) \\ sin\left(\frac{\pi}{3}\right) \end{pmatrix} b_2 = \begin{pmatrix} cos\left(\frac{2\pi}{3}\right) \\ sin\left(\frac{2\pi}{3}\right) \end{pmatrix} \tag{1}$$

$$z_i = b_i(F\vec{x} + \vec{x}_{offset}) \tag{2}$$

$$r_{GC} = max(0, cos(z_0) + cos(z_1) + cos(z_2)) \tag{3}$$

Here b0, b1 and b2 are the normal vectors for the cosine waves. 9 modules with constant orientation are used. F is the spatial frequency of the grids, starting at 0.0028*2π. The scales of successive grids are related by the scaling factor $\sqrt{2}$ [81]. The grid patterns of different cells in a module/scale are offset relative to each other [16], collectively covering the entire visual field evenly. For each grid scale 100 offsets are sampled uniformly along the principle axes of two adjacent equilateral triangles on the grid (i.e., the rhomboid made of 4 grid vertices). Thus the grid cell ensemble consists of 9 modules/scales with 100 cells each.

To calculate displacement vectors between locations encoded by grid cell population vectors we employ a distance-cell model, following Bush et al. [29] and Chen and Verguts [82]. Briefly, a given location on a 2D plane is uniquely represented by a set of grid cell phases (Figure 1B; [30]). Grid cells with appropriate phases in each module project to a single cell encoding the corresponding distance in each of four distance cells arrays, two for each of two non-co-linear axes. The two distance cell arrays belonging to the same axis project to two readout cells. One readout cell receives monotonically increasing weights from one distance cell array and monotonically decreasing weights from the other. For the second readout cell the connections increase/decrease in the opposite direction along the distance axis. The relative difference in firing rate between the two readout neurons encodes the displacement between start and goal locations along the given axis (Figure 1B). The connections between grid cells and distance cells are universal (i.e., not stimulus specific) and could be set up during development.

Since the resolution of images and grid maps is restricted to 440x440 pixels we allow for a small tolerance of 1% in pixel coordinates for translation vectors derived from vector computations. This allows for the compensation of small rounding errors for discrete pixel targets. It has been suggested that microsaccades might serve to tweak the alignment of the fovea [83]. Interestingly, Hafed and co-workers [84, 85] raise the possibility that a mechanism similar to the present model may also inform microsaccades, e.g., for fine feature discrimination ([83]; reviewed in [86]), or recognition at a distance. However, it remains to be seen if memory-guided microsaccades can occur at delays which exclude working memory sources.

Rather than eye-movement vectors, the location of the focus of attention might be calculated [87], although we do not distinguish the focus of attention from the target of fixation here. However, covert attention may be involved in recognition under restricted viewing conditions [11], which could have implications for critiques of scan-path theory.

Training phase

We assume that during learning of a new stimulus, salient locations on an image (e.g., regions of high contrast) are foveated via bottom-up attention, without knowledge of the stimulus identity, consistent with the typical eye-movements performed by human subjects as they encounter new faces [11]. This bottom-up processing would also include figure-ground segmentation. For simplicity this processing stage is not modeled and salient features are selected manually (9 per stimulus). In reality an analysis of the scene statistics may help select maximally de-correlated inputs [88, 89]. For face stimuli the selected locations include the corners of eyes, the tip or sides of the nose, corners of the lips, etc. That is, regions of the stimulus that exhibit strong gradients in contrast under most lighting conditions, and similar criteria are applied for scenes and objects. At each of the locations foveated during the training phase a cell coding for the attended feature (feature label cell, see Feature detection, below) is associated with the current grid cell population vector and with a single stimulus identity cell. Identity cells represent the abstract identity of a stimulus and are recruited on the fly during the first exposure to a stimulus. They might reside in face-, object- or scene specific neocortical area or the hippocampus (please see Discussion).

Feature detection and ambiguity

Feature detection is accomplished by banks of sensory neurons (with Gaussian tuning with regard to preferred gray-scale values) responding to the content of each pixel in the foveal array (Figure 2A,B). Preferred pixel values for Gaussian tuning curves of sensory cells are taken from blurred images during training (Mathworks, MATLAB function integral filter, range 5 pixels). This reflects (to a first approximation) the fact that a large variance in absolute pixel values would be encountered in reality, and the fact that stimuli of different identity may contain some similar-looking individual features (feature ambiguity). Depending on the content of the fovea a different subset of these sensory neurons is maximally active. During the training phase connections from these sensory cells to a given feature label cell are learned. That is, for a given foveal content the sum of all sensory cells maximally drives a given feature label cell, thus implementing a simple toy model of feature detection. The ambiguity of feature perception can lead to an incorrect initial hypothesis regarding the attended stimulus, i.e., an incorrect identity neuron being the most active. Such a hypothesis will produce saccades which do not take the fovea to expected features (Figure 2). As a consequence, sensory cells can drive many feature label cells due to incidental partial overlap between their preferred features and the content of the fovea. Those feature label cells impart some activity onto their associated identity neurons. That is a random subset of identity cells is weakly driven, leading to a flattening of the distribution of firing rates among stimulus identity cells. By contrast, when the output of the feature detectors primarily drives the correct feature label cells, and successive saccades confirm the initial hypothesis, the distribution of firing rates among stimulus identity cells becomes progressively more peaked. Alternatively to tracking mismatch events between predictions and sensory discriminations (see above) the absence of convergence to a specific hypothesis could be detected by the increased total amount of firing among stimulus identity cells.

Position Invariance

During learning, each feature of a given stimulus is anchored to the grid cell representation, and the relative locations of features encoded across all grids are mutually consistent. Paralleling experimental results [17, 20] we encode all stimuli in the same grid ensemble (as if anchored to a presentation screen). However, if connections from the most active feature label cell can re-align the grid cell ensemble (foveal array $\geq$ feature label cell $\geq$ grid cells) to the phases specific to a given stimulus, then recognition irrespective of the position of a stimulus in the field of view (position invariance) follows from the fact that the grid system encodes the relative (rather than absolute) locations of the features within a stimulus. This parallels observations from spatial navigation studies that show grid cell firing patterns are stable across visits to the same environment but shift coherently upon a change to the environment [30]. This predicts that grid cell phases will follow the position of the stimulus in the visual field, as suggested by recognition of illuminated faces in darkness (i.e., in the absence of environmental anchor points). Recognition irrespective of the position of a stimulus in the field of view (stimulus position invariance) follows from the fact that the grid system encodes the relative (rather than absolute) locations of the features within a stimulus. Associating each stimulus with a randomly shifted initial distribution of grid cell phases could reduce interference. However, because of the multitude of individual differences between stimuli (even those with a stereotypical layout of component features, e.g., in faces all eyes have roughly similar, but usually not identical, distances), all stimuli can be encoded in the same grid cell ensemble. Individual differences ensure that individual features map onto different grid cell population vectors.

Size Invariance

Pre-processing of visual inputs may conceivably generate a size invariant representation of stimuli prior to input to the system described here. However, the present model could accommodate size invariance. All circuit level models of grid cells require velocity inputs to update their firing (locomotor velocity during spatial navigation or eye-velocity in visual paradigms). The coupling between "neural space" (i.e., distance on the grid pattern) and self-motion has been shown to be plastic in spatial navigation paradigms [31, 90]. Hence we assume a given saccade length is subject to gain-modulation to give an appropriately scaled distance on the grid cell ensemble, and conversely, a given distance on the grid cell ensemble can be scaled to yield a saccade of appropriate length. This requires a change in gain with the estimated size (distance) of the stimulus. The estimation of stimulus size during recognition could reflect the size of the retinal image or ocular focus (compared to a memorized baseline), which is related to the distance and hence size of the stimulus (Thales theorem).

Note that the estimation of stimulus size during recognition does not necessitate prior recognition of a particular exemplar. The gain could be set dynamically against some baseline reference size (e.g., the size of the segmented retinal image at the time of the first encounter) to dynamically scale saccades.

QUANTIFICATION AND STATISTICAL ANALYSIS

Recognition rates were computed as the percentage of recognized stimuli. If a stimulus was not recognized within 10 resets (see Method Details) a fail was registered (labeled 'not rec.' in Figure 5).

DATA AND SOFTWARE AVAILABILITY

Code will be made available at https://github.com/bicanski

Current Biology, Volume *29*

Supplemental Information

A Computational Model of Visual Recognition Memory via Grid Cells

Andrej Bicanski and Neil Burgess

Supplementary Material

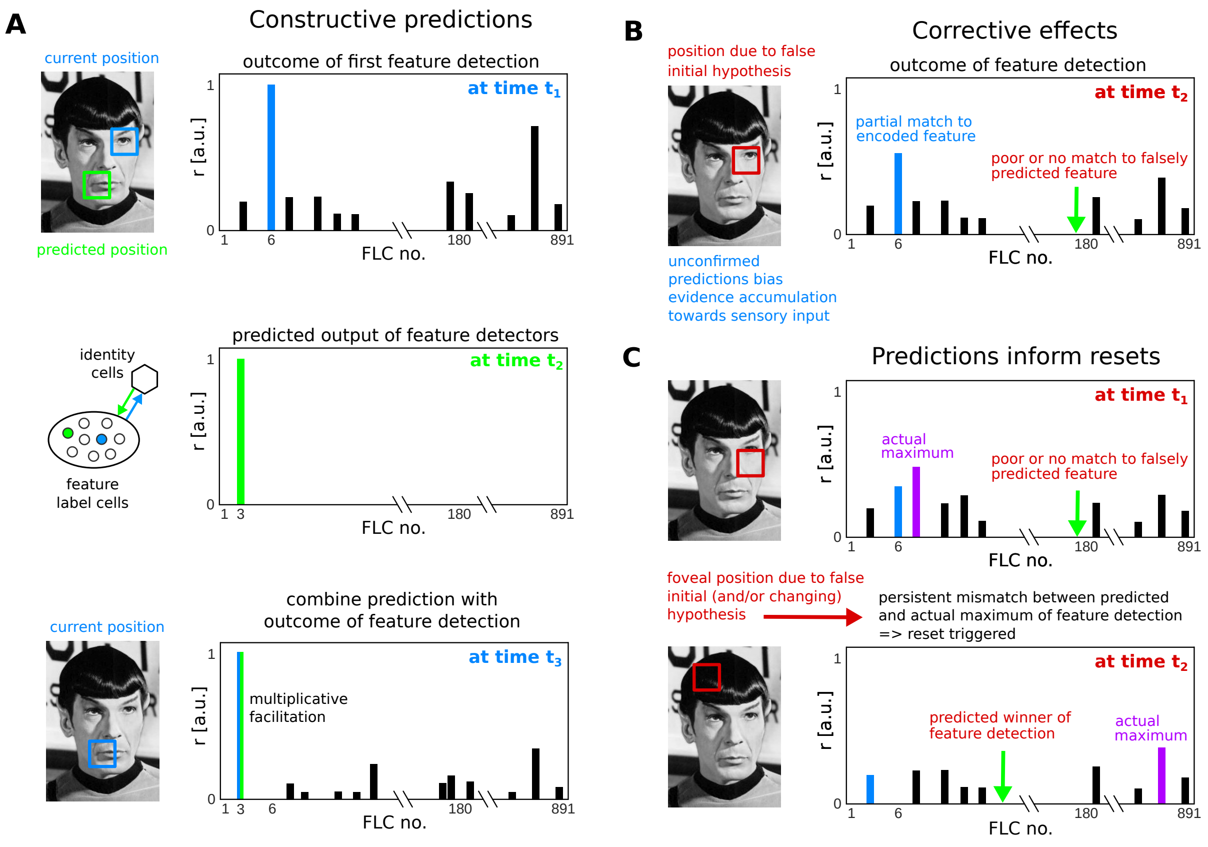

Figure S1: Detailed description of the effects of sensory predictions. (Related to Figure 1E)

A) The constructive nature of confirmed predictions. Top panel: The first foveated feature is evaluated by the feature detectors, which drive feature label cells (FLCs). The feature label cell representing the right eye of Mr. Spock is the strongest contender (blue bar in left sketch of FLC activity after thresholding), and it increments its associated stimulus identity cell (middle-right panel, blue arrow). The return projection (green arrow) selects the next saccade target randomly (green cell, target given by associated grid cell population vector, not shown). However, the firing of that FLC (green) also represents a prediction for the outcome of the next sensory discrimination once the fovea has located to the green square (top-left panel). Bottom panels: if there is any activity in the predicted FLC post saccade, its activity is boosted, allowing it to overcome interference from FLCs which received comparable or lower inputs. I.e. the leading hypothesis is prioritized if the competing inputs are of similar magnitude. **B)** Top panel: the lack of a confirmed prediction made due to an incorrect

initial hypothesis (green arrow in right panel) allows a partial feature match (blue bar) to accumulate evidence towards the correct hypothesis. **C)** If the predicted outcome of the feature discrimination (green arrow) and the maximally firing FLC (purple) do not match a mismatch event is registered. The red square in the left hand side panels indicates fixations to parts of the stimulus that will produce a poor sensory match due to being guided by an incorrect hypothesis. At the third registered mismatch event the model resets and starts a new recognition attempt, beginning with a different starting feature. Image credit; Mr. Spock: public domain image.

Table S1: All stimuli with picture credit (source URL) and license information. (Related to Results section and Figures 3,4,5)

In accordance with CellPress guidelines this table is supplied as a separate Excel file because it cannot fit onto three 8.5”x11” pages.

N_{std}	2.8
N_{Reset}	10
FLC2ID	0.4
Dec_{TH}	0.9
N_{fov}	61x61/31x31
N_{FPC}	9*99
N_{ID}	99
N_{SC}	61x61x256/31x31x256
N_{GC}	900 (100/module)
GC spatial frequencies	[0.0014 0.0211]
N_{DC}	440x4 (2 per axis)

Table S2: Main Parameters. (Related to detailed model description in STAR* Methods)

N_{std} sets the amount of initial pruning of feature label cell responses. It indicates the number of standard deviations the firing of a feature label cell has to reach above the mean of the entire population in order not to be silenced. N_{Reset} is the number of resets allowed. *FLC2ID* is the global connection strength of all feature label cells to their respective stimulus identity cells. The value of 0.4 signifies that if a single feature label cell is active (i.e. when the softmax across feature label cells has no effect), the firing of the associated stimulus identity cell is increased by 0.4 towards the decision threshold (Dec_{TH}). Cell numbers: Number of foveal cells (N_{fov}, second set of numbers indicates extent used for scaled-down stimuli), feature label cells (N_{FLC}), stimulus identity cells (N_{ID}), sensory cells (N_{SC}), grid cells (N_{GC}) across 9 modules across the interval (spatial frequencies geometrically spaced, multiplier √2), and distance cells (N_{DC}).
